# Bacterial pneumonia-induced shedding of epithelial heparan sulfate inhibits the bactericidal activity of cathelicidin in a murine model

**DOI:** 10.1152/ajplung.00178.2023

**Published:** 2023-12-19

**Authors:** Evan P. Zehr, Christopher L. Erzen, Kaori Oshima, Christophe J. Langouet-Astrie, Wells B. LaRiviere, Deling Shi, Fuming Zhang, Bruce D. McCollister, Samuel L. Windham, Alicia N. Rizzo, Julie A. Bastarache, Alexander R. Horswill, Eric P. Schmidt, Jakub M. Kwiecinski, James F. Colbert

**Affiliations:** ^1^Department of Medicine, University of Colorado School of Medicine, Aurora, Colorado, United States; ^2^Department of Chemical and Biological Engineering, Rensselaer Polytechnic Institute, Troy, New York, United States; ^3^Department of Medicine, Washington University School of Medicine, St. Louis, Missouri, United States; ^4^Department of Medicine, Massachusetts General Hospital, Boston, Massachusetts, United States; ^5^Department of Medicine, Vanderbilt University, Nashville, Tennessee, United States; ^6^Department of Medicine, Rocky Mountain Regional Veterans Affairs Medical Center, Aurora, Colorado, United States; ^7^Department of Microbiology, Jagiellonian University, Krakow, Poland

**Keywords:** antimicrobial peptides, cathelicidin, heparan sulfate, pneumonia, Staphylococcus aureus

## Abstract

Bacterial pneumonia is a common clinical syndrome leading to significant morbidity and mortality worldwide. In the current study, we investigate a novel, multidirectional relationship between the pulmonary epithelial glycocalyx and antimicrobial peptides in the setting of methicillin-resistant *Staphylococcus aureus* (MRSA) pneumonia. Using an in vivo pneumonia model, we demonstrate that highly sulfated heparan sulfate (HS) oligosaccharides are shed into the airspaces in response to MRSA pneumonia. In vitro, these HS oligosaccharides do not directly alter MRSA growth or gene transcription. However, in the presence of an antimicrobial peptide (cathelicidin), increasing concentrations of HS inhibit the bactericidal activity of cathelicidin against MRSA as well as other nosocomial pneumonia pathogens (*Klebsiella pneumoniae* and *Pseudomonas aeruginosa*) in a dose-dependent manner. Surface plasmon resonance shows avid binding between HS and cathelicidin with a dissociation constant of 0.13 μM. These findings highlight a complex relationship in which shedding of airspace HS may hamper host defenses against nosocomial infection via neutralization of antimicrobial peptides. These findings may inform future investigation into novel therapeutic targets designed to restore local innate immune function in patients suffering from primary bacterial pneumonia.

**NEW & NOTEWORTHY** Primary *Staphylococcus aureus* pneumonia causes pulmonary epithelial heparan sulfate (HS) shedding into the airspace. These highly sulfated HS fragments do not alter bacterial growth or transcription, but directly bind with host antimicrobial peptides and inhibit the bactericidal activity of these cationic polypeptides. These findings highlight a complex local interaction between the pulmonary epithelial glycocalyx and antimicrobial peptides in the setting of bacterial pneumonia.

## Graphical abstract

**Figure:**
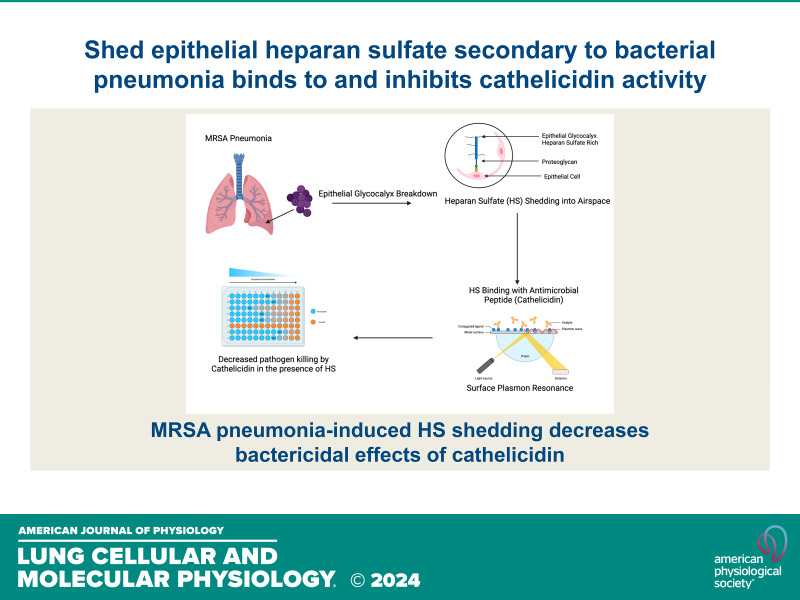

## INTRODUCTION

The clinical syndrome of pneumonia is the leading cause of mortality due to infectious etiologies (1). Among critically ill patients, *Staphylococcus aureus*—specifically methicillin-resistant *Staphylococcus aureus* (MRSA)—is the most common gram-positive pneumonia-causing microorganism (2). However, the pathophysiological mechanisms underlying predisposition to the development of Staphylococcal pneumonia are incompletely understood. In the current study, we investigate the implications of interactions among MRSA, the pulmonary epithelial glycocalyx, and antimicrobial peptides (AMPs) as a novel mechanistic determinant of pneumonia pathology.

The pulmonary epithelial glycocalyx is a heparan sulfate (HS)-enriched layer lining the alveolar lumen. HS is a sulfated glycosaminoglycan that is anchored to the alveolar epithelial surface by transmembrane proteoglycans. The anionic charge afforded by sulfation allows HS to specifically bind and shape the function of cationic proteins (3, 4). Our group has previously shown that direct lung injury induced by viral infection, lipopolysaccharide, and bleomycin exposure results in the shedding of pulmonary epithelial HS into the airspace (5–7). This shedding not only results in increased lung permeability and surfactant dysfunction but also may exert unexplored actions of the biologically active fragments that can linger within the airspaces for days to weeks (5, 8).

The goal of this study is to investigate the interactions between shed epithelial HS oligosaccharides, the local host immune response, and bacterial pathogens during primary bacterial pneumonia. We pay specific attention to the likely interaction of anionic HS fragments with cationic antimicrobial peptides (AMPs) such as cathelicidins (9, 10). These polypeptides are important innate immune mediators and have previously been demonstrated to be present in the pulmonary airspace during experimental pneumonia (11) and human acute respiratory distress syndrome (ARDS) (12). The complex interplay between shed pulmonary HS, AMPs, and bacterial pathogens as it relates to primary bacterial pneumonia and susceptibility to secondary pneumonia is unclear. Using complementary in vivo and in vitro models, we here investigate our overall hypothesis that bacterial pneumonia leads to pulmonary epithelial HS shedding, altering the bactericidal effect of AMPs through direct binding of HS.

## MATERIALS AND METHODS

### Study Approval and Design

Animal experiments were conducted under an approved University of Colorado Institutional Animal Care and Use Committee (IACUC) protocol and in accordance with the Animal Research: Reporting of In Vivo Experiments (ARRIVE) guidelines (13). For in vivo experiments, sample size was based on prior work by our group and a power calculation suggested that five biological replicates would result in detectable differences in shed HS (5–7). Quantification of HS via mass spectrometry was performed by colleagues blinded to the treatment groups (IT MRSA vs. saline control). We included female mice in the control group to address the potential confounder of biologic sex as a mediator of epithelial glycocalyx shedding (8). Due to the use of potentially infectious agents, an Institutional Biosafety Committee (IBC) protocol was additionally approved and followed.

### Intratracheal Pneumonia Model

Eight- to ten-wk-old C57BL6 male mice (Jackson Laboratories) were anesthetized with inhaled isoflurane (3–5%) before intratracheal instillation of either MRSA (1 × 10^8^ CFU/30 µL) or 30 µL of saline control. Twenty-four h after instillation, bronchoalveolar lavage (BAL) was performed three times, each with 1 mL of sterile PBS. BAL fluid was collected and centrifuged at 1,000 *g* for 5 min and supernatant was stored for subsequent analyses. USA300 (LAC) MRSA strain (AH1263) was utilized as the infectious insult and concentration was determined via optical density as described previously (7).

### Heat and Moisture Exchanger Filter Collection

We collected airspace fluid from patients with pneumonia-induced respiratory failure using heat and moisture exchanger (HME) filters, an airway humidification device used in the standard respiratory care of mechanically ventilated patients. HME filters were collected at Vanderbilt University Medical Center Medical intensive care unit (ICU) under an approved Institutional Review Board (IRB) protocol as previously described (8). Participants were enrolled between November 2017 and July 2020. A fresh HME filter was placed in the participants ventilator circuit and subsequently collected after a 4-h dwell time per previously published protocols (14).

### Mass Spectrometry Quantification of HS Concentrations

Mass spectrometry was used to directly quantify HS in BAL fluid as described previously (5, 6). In brief, samples were washed and desalted using a 3-kDa molecular mass cutoff spin column (Millipore). HS was then digested with recombinant heparin lyases (I, II, III) in digestion buffer (50 mM ammonium acetate containing 2 mM calcium chloride adjusted to pH 7.0) at 37°C for 4 h. Enzymes were then removed by centrifugation. Disaccharide products were lyophilized and labeled with 2-aminoacridone. UPLC-tandem mass spectrometry was performed on a Dionex LC system and an AB Sciex Qtrap 5500 with multiple reaction monitoring (MRM) mode. Based on prior work, a correction factor of 37.92 was uniformly multiplied to the concentration of HS recovered in the BAL to estimate the concentration of HS (5).

### Bacterial Growth Curves

Growth curves were performed in Dulbecco’s modified Eagle’s medium (DMEM) for two different MRSA strains (AH1263, USA300 [LAC] + AH843, USA400 [MW2]) as previously described (15). Conditions for each strain included water (control), full-length heparin, *N*-desulfated re-*N*-acetylated heparin (nonanticoagulant, desulfated heparin), and 3 lengths of heparin oligosaccharides [degrees of polymerization (DP); DP6, DP10, and DP20].

### Surface Plasmon Resonance and Binding Kinetics between AMP and HS

Surface plasmon resonance (SPR) provides real-time and label-free measurements on binding kinetics and affinity for biomolecular interactions (16). Murine cathelicidin-related antimicrobial peptide (mCRAMP) was purchased from AnaSpec. Porcine intestinal heparin with an average molecular mass of 15 kDa and polydispersity of 1.4 was purchased from Celsus Laboratories (Cincinnati, OH). Two milligrams of heparin and 2 mg of amine-PEG3-Biotin (Thermo Scientific, Waltham, MA) were mixed with 10 mg of NaCNBH3. The initial reaction was carried out at 70°C for 24 h, and then a further 10 mg of NaCNBH3 was added to continue running the reaction for another 24 h. After completion of the reaction, the mixture was desalted with a spin column (3,000 molecular weight cutoff). Biotinylated heparin was freeze-dried for chip preparation. The biotinylated heparin was immobilized to a streptavidin (SA) chip based on the manufacturer’s protocol. In brief, 20 μL solution of the heparin-biotin conjugate (0.1 mg/mL) in HBS-EP + buffer (0.01 M 4-(2-hydroxyethyl)-1-piperazineethanesulfonic acid, 0.15 M NaCl, 3 mM ethylenediaminetetraacetic acid, 0.05% surfactant P20, pH 7.4) was injected over flow cell 2 (FC2), flow cell 3 (FC3), and flow cell 4 (FC4) of the SA chips at a flow rate of 10 μL/min. The successful immobilization of heparin was confirmed by the observation of an ∼200 resonance unit (RU) increase in the sensor chip. The control flow cell (FC1) was prepared by 1-min injection with saturated biotin (no HS immobilized, serving as a negative control). mCRAMP was diluted in HBS-EP buffer. Different dilutions of protein samples were injected at a flow rate of 30 µL/min. At the end of the sample injection, the same buffer was flowed over the sensor surface to facilitate dissociation. After a 3-min dissociation time, the sensor surface was regenerated by injecting with 30 µL of 2 M NaCl to get fully regenerated surface. The response was monitored as a function of time (sensorgram) at 25°C.

### Bacterial RNA Isolation and Sequencing

USA 400 (MW2) MRSA strain (AH843) was grown in Tryptic Soy Broth (TSB) for 18–24 h at 37°C with 200 rpm of shaking. Bacterial suspensions were diluted to 1:1,000 and were cultured for 3 or 10 h at 37°C with 1,000 rpm of shaking in the presence of saline or unfractionated heparin (2 μg/mL). Bacterial suspensions were centrifuged and resuspended in cold PBS to an optical density of OD600, and RNA was isolated using RNeasy Mini Kit (Qiagen) following the manufacturer’s recommendations followed by DNase treatment using the TURBO DNA-free Kit (Invitrogen). RNA samples were submitted to the University of Colorado Denver Genomics and Microarray Shared Resource for Bioanalyzer quality control analysis (Agilent) and Illumina sequencing.

### Transcriptomic Analyses

Raw sequencing reads in FASTQ format were adapter trimmed, aligned, and annotated to the USA400 reference genome (NC_007795.1) using Qiagen CLC Genomics Workbench default settings (Version 21.0.5): mismatch cost, 2; insertion and deletion cost, 3; length and similarity fraction, 0.8. Normalization and differential expression calculations of uniquely mapped mouse or bacterial transcripts were performed using R package DESeq2 (Version 1.34.0, RRID:SCR_015687) (17). Differentially expressed genes were filtered based on two criteria: *1*) an absolute log2 fold change cutoff and *2*) Benjamini–Hochberg false discovery rate (FDR) cutoff. MRSA RNA sequencing data have been deposited to the National Center for Biotechnology Information Gene Expression Omnibus (GEO) with accession number GSE231407.

### Minimum Inhibitory Concentration Quantification

The minimum inhibitory concentrations (MICs) of three bacterial pneumonia strains (*Klebsiella pneumoniae* NTUH-K2044, *Pseudomonas aeruginosa* PAO1, and *Staphylococcus aureus* USA 400 MW2 [AH843]) to mCRAMP were measured in the presence of varying concentrations of HS as previously described (18). In brief, the three bacterial strains were prepared based on optical density measurements at 600 nm and exposed to 6 [2-fold] mCRAMP concentrations (128–4 µg/mL) and 4 [10-fold] HS concentrations (200–0.2 µg/mL) for a total of 24 experimental conditions. A modified radial diffusion assay was used with zone of inhibition diameter measurement at the 24-h timepoint. To calculate the minimal inhibitory concentration (MIC), the diameters calculated above were converted to units (10 U = 1 mm). Data points were plotted (*x* = [AMP], *y* = zone diameter). A line of best fit was calculated using a logarithmic regression, with the *x*-intercept of these lines representing the MIC of mCRAMP.

### Statistical Analysis

**Figure 1.**
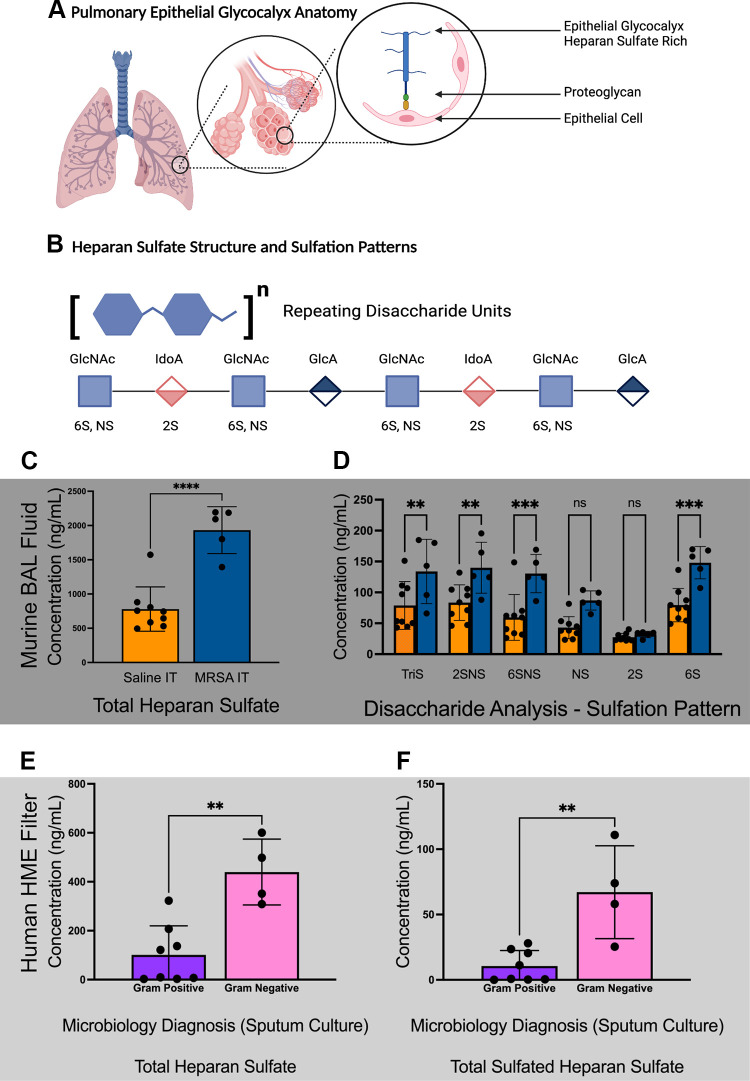
Bacterial pneumonia induces shedding of sulfated heparan sulfate (HS) into the airspace. *A*: graphic representation of the pulmonary epithelial glycocalyx. Glycosaminoglycans (GAGs), such as heparan sulfate (HS), are tethered to epithelial cells via a proteoglycan (PG). Together, these HS-proteoglycans, along with other GAG-PG, form the pulmonary epithelial glycocalyx. *B*: heparan sulfate consists of repeating units of glucosamine plus glucuronic acid or its epimer iduronic acid that are covalently linked to a PG. Following synthesis, the HS chain can undergo modification including epimerization of glucuronic acid, *N*-sulfation of glucosamine (denoted NS), sulfation at glucosamine (6S), and sulfation of iduronic acid (2S). These reactions occur sparsely across the length of the GAG and within each disaccharide unit, resulting in 7 possible sulfation patterns per disaccharide. *C* and *D*: murine MRSA pneumonia in vivo model (dark gray shading). *C*: total HS shedding into BAL fluid quantified via mass spectrometry after intratracheal instillation of MRSA compared with control saline instillation. *D*: quantification of individual disaccharide sulfation patterns after intratracheal MRSA pneumonia. *n* = 9 for saline control animals, *n* = 5 for MRSA pneumonia-treated animals. *E* and *F*: human HME filter HS quantification broken down by clinical microbiology data; gram-positive organism vs. gram-negative organism (light gray shading). *E*: total HME filter HS quantified via mass spectrometry. *F*: HME filter-sulfated HS quantified via mass spectrometry. *n =* 8 for microbiologically confirmed gram-positive pneumonia cases, *n =* 4 for microbiologically confirmed gram-negative pneumonia cases. Data are presented as means ± standard deviation. Statistical testing (unpaired, two-tailed *t* test) performed for between-group comparisons. **P < 0.01; ***P < 0.001; ****P <0.0001. BAL, bronchoalveolar lavage; HME, heat and moisture exchanger; MRSA, methicillin-resistant *Staphylococcus aureus*. [Images A and B created with a licensed version of BioRender.com.]

All statistical analyses were performed using GraphPad Prism (Version 9.5.0). Datasets were assumed to have normal distribution and variance. Statistical significance was defined as a *P* value of <0.05. Unpaired, two-tailed *t* test was used for statistical testing between groups (Fig. 1), whereas datasets with multiple concentrations were measured by ANOVA with Tukey’s post hoc analysis and adjustment for multiple comparisons (Fig. 4).

## RESULTS

### Bacterial Pneumonia Induces Shedding of Sulfated HS into the Airspace

To determine whether primary bacterial pneumonia induces pulmonary epithelial HS shedding (Fig. 1*A*), we used our previously published murine model of primary MRSA pneumonia and quantified total HS shedding and the frequency of N-sulfation (NS), 2-sulfation (2S), and 6-sulfation (6S) via mass spectrometry (Fig. 1*B*). About 24 h after intratracheal instillation of MRSA, we observed a significant increase (*P* < 0.01) of heparan sulfate (*n* = 5, mean = 1,930 ng/mL) in the airspace lining fluid as compared with saline control animals (*n* = 9, mean = 780 ng/mL; Fig. 1*C*). The time course and amount of HS are similar to our previous findings using alternative infectious and noninfectious insults (5–7). The absolute concentrations of the various sulfated disaccharides are presented in Fig. 1*D* and demonstrate an increased abundance of sulfated HS from MRSA-treated animals. These differences were predominantly driven by an increase in multisulfated disaccharides (Tri-S, 2SNS, 6SNS) and 6-sulfated disaccharides. We used complementary human HME filter samples to assess the role of gram-positive versus gram-negative bacterial etiologies as a contributor to epithelial HS shedding. We demonstrate higher HS levels in patients with gram-negative pneumonia compared with that of gram-positive pneumonia (Fig. 1*E*). In addition, gram-negative pneumonia led to higher concentrations of sulfated HS in HME filters (Fig. 1*F*). Our findings suggest that lung injury due to bacterial pneumonia leads to an acute shedding of epithelial pulmonary HS into the airspace, which is enriched in sulfated—and likely biologically active—HS oligosaccharides.

### Shed HS Does Not Impact MRSA Growth or Gene Transcription

**Figure 2.**
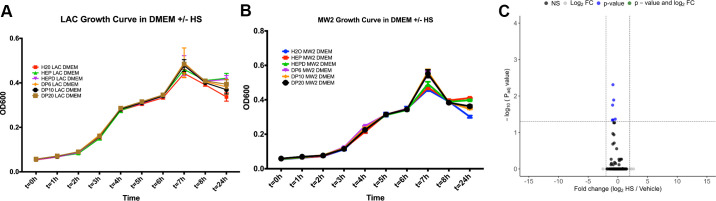
Shed HS does not impact MRSA growth or gene transcription. Growth curves for USA300 LAC MRSA strain (*A*) and USA400 MW2 MRSA strain (*B*) in the presence of HS. H_2_O, water control; HEP, full-length HS; HEPD, *N*-desulfated, re-*N*-acetylated HS (nonanticoagulant, desulfated HS); DP6, HS 6 monosaccharides in length; DP10, HS 10 monosaccharides in length; DP20, HS 20 monosaccharides in length. *n* = 6 biological replicates for each growth curve. *C*: bulk RNA sequencing volcano plot demonstrating zero differentially expressed genes with our cutoff values for significance (log2FC > |2|, *P*.adj < 0.05) between MRSA treated with HS or vehicle control. DP, degrees of polymerization; HS, heparan sulfate; MRSA, methicillin-resistant *Staphylococcus aureus*.

Our in vivo findings prompted us to investigate whether shed HS directly inhibited growth or altered the transcriptomic response of the inciting bacterial pathogen. We exposed both USA300 and USA400 MRSA strains to various sizes and sulfation patterns of HS to assess growth alterations. Importantly, there was no change in MRSA growth regardless of HS sulfation [using unsulfated (*N*-desulfated re-*N*-acetylated) heparin as a control] or length of HS oligomer (DP6, DP10, DP20) (Fig. 2, *A* and *B*). To assess bacterial transcriptomic changes in response to this altered environment, we used an in vitro model and bulk RNA sequencing. USA 400 MRSA was exposed to either diluent or HS to assess differential messenger RNA response. Under these conditions, no genes were differentially expressed to meet our cutoff values for significance (log2FC > |2|, *P*.adj < 0.05) as demonstrated in the volcano plot (Fig. 2*C*). These experiments suggest that shed HS—unlike other constituents of the injured lung milieu—is not sufficient to inhibit bacterial growth or induce altered MRSA transcription patterns (7).

### Surface Plasmon Resonance Demonstrates Direct Binding of HS and mCRAMP

**Figure 3.**
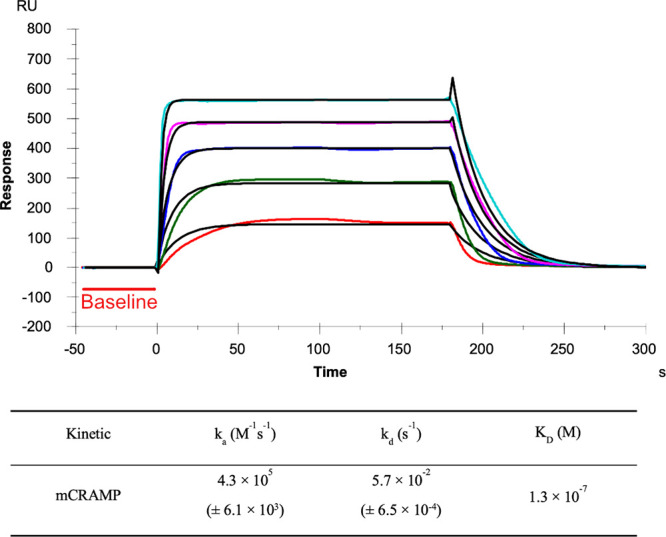
Surface plasmon resonance demonstrates direct binding of HS and mCRAMP. SPR sensorgrams of mCRAMP binding with heparin are displayed. Concentrations of mCRAMP (from top to bottom): 1,000, 500, 250, 125, and 63 nM, respectively. The black curves are the fitting curves using models from T200 Evaluate software 3.2. A summary of kinetic data of mCRAMP binding with heparin is shown in the table. The data with (±) in parentheses are the standard deviations (SDs) from global fitting of 5 injections. HS, heparan sulfate; mCRAMP, murine cathelicidin-related antimicrobial peptide; SPR, surface plasmon resonance; RU, resonance unit.

Owing to the absence of obvious pathogen growth or transcription changes in response to HS, we sought to evaluate whether HS has effect on host innate immune mediators. Prior groups have demonstrated indirect evidence that HS binds AMPs, likely via electrostatic interaction (19). To address this question directly, we used surface plasmon resonance (SPR) to quantify binding of HS with mCRAMP (Fig. 3). We found strong binding between these two molecules (dissociation constant of 0.13 μM), suggesting that HS-mCRAMP interaction was likely to occur in vivo and may shape the host response to bacterial infection.

### HS Inhibits mCRAMP Bactericidal Activity against Nosocomial Pneumonia Pathogens

**Figure 4.**
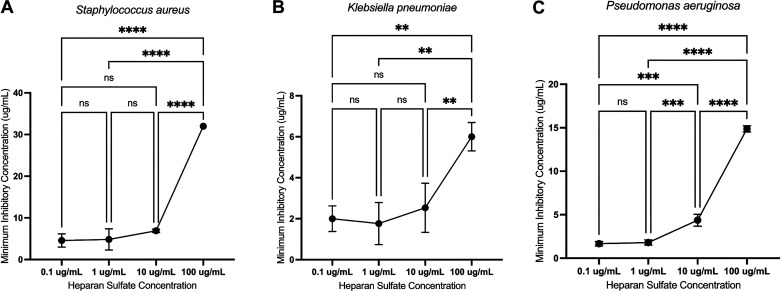
HS inhibits mCRAMP bactericidal activity against nosocomial pneumonia pathogens. Minimum inhibitory concentration (MIC) of mCRAMP in the presence of varying concentrations of HS measured via a modified radial diffusion assay for three different bacterial pathogens: *Staphylococcus aureus* (*A*), *Klebsiella pneumoniae* (*B*), and *Pseudomonas aeruginosa* (*C*). *n* = 3 experimental replicates per condition (48 measurements per experimental replicate). There was no inhibition of growth for any of the 3 bacterial species in the presence of HS alone (without mCRAMP). Ordinary one-way ANOVA performed with Tukey’s multiple comparison test to compare the means of all groups. **P < 0.01; ***P < 0.001; ****P < 0.0001. HS, heparan sulfate; mCRAMP, murine cathelicidin-related antimicrobial peptide; ns, not significant.

Given our observation that HS avidly binds mCRAMP, we sought to determine whether HS functionally inhibited the antibacterial activity of AMPs. We expanded our analysis to include other common nosocomial pneumonia pathogens in addition to MRSA: *Klebsiella pneumoniae* and *Pseudomonas aeruginosa*. We used a modified radial diffusion assay to calculate the MIC of mCRAMP against these pathogens (Fig. 4). For all three bacterial species, there were significant changes in minimum inhibitory concentration (MIC) with increasing concentrations of HS. MICs were significantly higher (indicating decreased bactericidal effects of mCRAMP) at 100 μg/mL of HS (Fig. 4, *A*–*C*). These findings highlight the complexity of this interaction as increasing HS concentrations had no individual direct effect on MRSA growth (Fig. 2*A*). However, with the addition of a known bactericidal AMP (mCRAMP), HS plays a pivotal role in altering the pathogen killing properties of this polypeptide.

## DISCUSSION

These findings call attention to a complex, multidirectional relationship among shed pulmonary epithelial HS oligosaccharides, the local host immune response, and bacterial pathogens during pneumonia. The mass spectrometry quantification of airspace HS after bacterial pneumonia (both murine MRSA-induced and human HME filter) is consistent with our group’s work on other models of lung injury, suggesting that various direct lung injury modalities may lead to a similar glycocalyx response (5–7). These data are also supportive of prior studies, demonstrating that models of *P. aeruginosa* and *S. aureus* pneumonia result in shedding of the HS-binding proteoglycan, syndecan-1, into the airspace (19, 20). Although the presence of syndecan-1 after bacterial pneumonia suggests glycocalyx shedding, the current study adds to this body of literature by measuring not only the protein component but also the confirmation of glycocalyx breakdown by direct quantification of shed total HS and stratification of sulfated disaccharides. Specific sulfation patterns of HS disaccharides are hypothesized to play an important role as determinants of pneumonia pathophysiology and should be investigated in future studies.

The translational applicability of these findings applies to multiple clinical syndromes in addition to bacterial pneumonia. Our data suggest that there may be a unifying pulmonary response to direct injury whether via bacterial infection, viral infection, sterile insult (i.e., LPS), or drug-induced toxicity. Conceptually, the shedding of glycosaminoglycans in response to primary injury is thought to be physiological and protective. However, these fragments may facilitate unintended consequences—such as predisposition to bacterial pneumonia—through competitive interactions with cationic AMPs. Mechanistic awareness of this susceptibility to bacterial infection could facilitate therapeutic intervention strategies and impactful changes in the management of patients with a multitude of direct lung injury syndromes. In addition, similar interactions have been exhibited in models of cystic fibrosis, implying that an understanding of this interplay may hold significance in the management of chronic respiratory illnesses as well (21).

This study has several strengths that increase our understanding of the host-pathogen interactions occurring in pneumonia pathogenesis. Interestingly, despite our prior work demonstrating increased virulence potential of MRSA in the injured lung environment, this phenotype is not explained by transcriptional changes in response to direct sensing of HS (7). Instead, we propose a construct where pathogen-induced glycocalyx shedding leads to a deleterious host-host interaction (binding between HS and AMPs). To our knowledge, these data are the first to establish pulmonary epithelial HS shedding in response to primary bacterial pneumonia through direct quantification rather than proteoglycan-targeted assays. In addition, although hypothesized by other groups previously, this work confirms direct binding of anionic HS and cationic AMPs (mCRAMP) using surface plasmon resonance. Our in vitro quantification of the antibacterial consequences of this dose-response interaction on multiple nosocomial pneumonia pathogens builds on prior studies and adds to the clinic relevance of these findings (21). It is important to note that relatively small changes in pathogen-specific MICs to antibiotics can have a large impact on treatment outcomes. We propose here that the changes observed in antimicrobial peptide MICs in the presence of HS may have a similar impact on clinical pneumonia outcomes.

The primary limitation of this study is the reductionist in vitro investigation into a more complex in vivo system that may not identify all in vivo mediators. However, we believe the knowledge gained through these approaches will inform a more comprehensive understanding in the future. An additional consideration is the use of the murine analog (mCRAMP) for experimentation rather than human cathelicidin (LL37). These two polypeptides are thought to have very similar size, charge, and structure and are often used interchangeably. However, there are examples of differential function and these findings would need to be confirmed with the human analog before further clinical investigation (22). Finally, it is unknown whether the concentrations of both HS and mCRAMP used for our in vitro experiments are physiologically relevant. There are many contributors to local concentrations of HS at the anatomic site of injury that are not fully quantified by BAL or HME filter measurement. In addition, the anatomic concentration of cathelicidins is dependent on delivery by circulating neutrophils (23), which is a dynamic process during pneumonia physiology.

In conclusion, this study highlights a previously unexplored interaction between pulmonary epithelial HS and cathelicidin in response to bacterial pneumonia. The rigorous confirmation of HS shedding after MRSA pneumonia, direct binding between HS and mCRAMP, and decreased bactericidal activity as a result of this binding form a framework for future mechanistic investigation into the clinical implications of these findings.

## ETHICAL APPROVALS

Animal experiments were conducted under approved University of Colorado Institutional Animal Care and Use Committee (IACUC) and Institutional Biosafety Committee (IBC) protocols.

## DATA AVAILABILITY

All data presented are available in the manuscript and accompanying figures. RNA sequencing data have been deposited to the National Center for Biotechnology Information Gene Expression Omnibus (GEO) with accession number GSE231407.

## GRANTS

This work was supported by NIH Grants K08AG061144 (J.F.C.), R01AI153185 (A.R.H.), and R01HL125371 (E.P.S.).

## DISCLOSURES

Julie Bastarache and Eric Schmidt are editors of *American Journal of Physiology-Lung Cellular and Molecular Physiology* and were not involved and did not have access to information regarding the peer-review process or final disposition of this article. An alternate editor oversaw the peer-review and decision-making process for this article. None of the other authors has any conflicts of interest, financial or otherwise, to disclose.

## AUTHOR CONTRIBUTIONS

E.P.S. and J.F.C. conceived and designed research; E.P.Z., C.L.E., K.O., C.J.L.-A., W.B.L., D.S., F.Z., S.L.W., A.N.R., J.A.B., J.M.K., and J.F.C. performed experiments; E.P.Z., C.L.E., K.O., C.J.L.-A., W.B.L., D.S., F.Z., B.D.M., S.L.W., A.N.R., J.A.B., J.M.K., and J.F.C. analyzed data; E.P.Z., C.L.E., K.O., C.J.L.-A., W.B.L., D.S., F.Z., B.D.M., S.L.W., A.N.R., J.A.B., A.R.H., E.P.S., J.M.K., and J.F.C. interpreted results of experiments; E.P.Z., J.F.C., C.J.L., D.S., and F.Z. prepared figures; E.P.Z., E.P.S., and J.F.C. drafted manuscript; E.P.Z., C.L.E., K.O., C.J.L.-A., W.B.L., F.Z., B.D.M., S.L.W., A.N.R., J.A.B., A.R.H., E.P.S., J.M.K., and J.F.C. edited and revised manuscript; E.P.Z., C.L.E., K.O., C.J.L.-A., W.B.L., D.S., F.Z., B.D.M., S.L.W., A.N.R., J.A.B., A.R.H., E.P.S., J.M.K., and J.F.C. approved final version of manuscript.
